# Fluid Balance, Sweat Na^+^ Losses and Carbohydrate Intake in Elite Male Soccer Players Training in Cool Environments

**DOI:** 10.3390/nu18203459

**Published:** 2026-10-21

**Authors:** Vlad Sabou, Heather Z. Macrae, Kevin Mulholland, Daniel Webber, Liam R. Brown, Rebecca K. Randell, Caroline A. Tarnowski, Lewis James, Ian Rollo

**Affiliations:** 1 Gatorade Sports Science Institute, PepsiCo Life Sciences, Global R&D, Leicestershire LE4 1ET, UK; 2 School of Sport, Exercise and Health Sciences, Loughborough University, Leicestershire LE11 3TU, UK; 3 Wrexham Association Football Club, Mold Road, Wrexham LL11 2AH, UK

**Keywords:** hydration, soccer, sweat loss, fluid intake, cool temperature, carbohydrate, sodium

## Abstract

Background/Objectives: Hypohydration increases physiological strain and reduces physical and technical soccer performance. Nonetheless, there are limited data on fluid balance responses in elite soccer players training in cool environments. This study investigated sweat and fluid/carbohydrate intake responses in elite male professional soccer players training at a high intensity in cool environments. Methods: Fluid/sodium (Na^+^) losses and *ad libitum* carbohydrate/fluid intake of thirty-six elite male soccer players were measured as part of four training sessions, with each player completing the measurements once. Data were collected under the following conditions classified by ambient temperature (11.3 ± 2.1 °C), wet bulb globe temperature (9.8 ± 3.0 °C), and relative humidity (76.8 ± 10.2%). Mean duration of training sessions was 70 ± 4 min and included soccer-specific training, with players covering a mean total distance of 7054 ± 654 m and 104 ± 9 (m·min^−1^). Before and after exercise, players were weighed in minimal clothing. During training breaks, players had *ad libitum* access to carbohydrate beverages and water. Sweat [Na^+^] (mmol·L^−1^) was measured by absorbent patches positioned on the upper back. Results: Two out of eleven players displayed a USG score indicative of ‘minimal hypohydration’ (1.022 and 1.022, respectively). Seventeen percent of players experienced hypohydration > 2% of pre-exercise body mass. Mean sweat rate was 1.5 ± 0.4 L·h^−1^, with individual sweat rates ranging from 0.4–2.2 L·h^−1^ (outfield players, *n* = 33). Goalkeepers mean sweat rate was 0.7 ± 0.2 L·h^−1^ (range 0.6–0.9 L·h^−1^, *n* = 3). Sweat [Na^+^] also varied substantially between players (range 21–49 mmol·L^−1^), with a mean sweat [Na^+^] of 35 ± 6.8 mmol·L^−1^. There was a significant positive correlation between sodium chloride loss and intensity of play (m·min^−1^) covered during training (r_s_ = 0.368, *p* = 0.049). Carbohydrate ingestion during training varied between individuals (range 0–25 g·h^−1^). Conclusions: Sweat rate and sweat sodium chloride loss varies in elite soccer players during high-intensity training in cool environments. Hypohydration exceeded 2% pre-exercise body mass in 17% of players. The *ad libitum* carbohydrate intake of the players was below recommendations given the intensity and duration of the training sessions.

## 1. Introduction

High-intensity intermittent running completed during soccer matches and training causes an elevation in core temperature (to 39–40 °C) [1]. Thermoregulatory responses include an increased skin blood flow and the onset of sweating for evaporative heat loss [2]. When sweat losses are not matched by fluid intake during exercise, hypohydration occurs. Hypohydration equal to, or in excess of, 2–3% of pre-exercise body mass can negatively impact cardiovascular [3], thermoregulatory [4] and mental [5] functions, which contributes to fatigue [6]. Repeated sprints and high-intensity running also lead to marked decreases in muscle glycogen concentrations [7]. Reduced glycogen availability also contributes to fatigue and is associated with a reduction in sprint performance, skill execution and distance covered at the end of a soccer match [7,8]. Furthermore, dehydration can enhance glycogen depletion during a soccer match due to an increase in carbohydrate utilisation when exercising in a dehydrated state [9].

Hypohydration of various magnitudes is reported during both elite soccer training (−1.6–3.5% pre-training body weight) [10,11,12,13] and matches (−1.8–2.6% pre-match body weight) [14]. When hypohydration exceeds 2–3% of pre-exercise body mass, technical [15] and physical [16] soccer performance can be negatively impacted. However, not all studies report impaired soccer performance in tandem with hypohydration in a temperate environment [17]. There is also a large inter-individual variability in the sweat rates of elite soccer players (0.5–2.5 L h^−1^) [11,12,13] This is because environmental conditions, training intensity and body size represent the main factors influencing sweat rate [11,13].

The majority of studies have investigated sweat rates of elite soccer players during training in the heat [11,12,18,19]. Studies reporting sweat response in cool environments [10,11,13] report lower mean sweat rates (1–1.2 L·h^−1^) versus hot environments (1.4–1.7 L·h^−1^) [11,13]. Nonetheless, hypohydration can still occur when training in cool weather (−1.62 ± 0.55%, range −0.87–2.55% of pre-training body mass) [9]. Sweat [Na^+^] and sodium loss also vary between soccer players completing the same training in cool environments [10,11,13]. The previous three studies that investigated sweat rates, sweat [Na^+^] and sodium loss in cool environments were all completed several competitional seasons ago (<2020–2021). Since then, the physical demands of elite Premier League soccer match play have increased, particularly regarding high-speed running, high-intensity distance and sprint distance [20]. However, the changes in training demands of soccer are unknown. There is likely significant variation in training intensity based on the phase of the season, competitive schedule, and individual coach training philosophy. Nonetheless, given the importance of running intensity and volume on match day performance, clubs continue track training running metrics using Global Positioning Systems (GPS). Given that exercise intensity represents an important factor that can impact both sweat rate and sodium loss [13], contemporary observations of training intensity that also characterize the fluid balance of elite soccer players training in cool environments are warranted.

There are large inter-individual differences in fluid intake in soccer players training in cool environments [10,11,13]. Soccer players consume 44–1050 mL of fluid per hour [10,11,13]. It appears that when training in cool environments, soccer players display highly variable *ad libitum* hydration strategies, similarly to when training and competing in the heat [21].

Carbohydrate intake during soccer training is of interest given the potential ergogenic effects on various aspects of soccer-specific performance [8]. In addition, consuming carbohydrates during training sessions completed at match-play intensities could help ‘train’ the gut, although this finding was previously shown in endurance runners only [22]. When practiced repeatedly long-term, this strategy may lead to improved tolerance to carbohydrates and reduced gastrointestinal distress during soccer matches [23]. Alternatively, soccer players may also purposefully choose not to consume carbohydrates during training to facilitate individual goals (e.g., manipulation of body composition) and to meet daily carbohydrate targets [23]. In addition, players may seek to adopt a “train low” approach to training to promote fat oxidation adaptation [24], or this practice may simply reflect the increased popularity of ketogenic diets in soccer [25]. To date, only one study has reported on carbohydrate intake during soccer training completed in cool environments [11]. Carbohydrate intake was similar during both low and high intensity sessions—11 g and 12 g per hour, respectively. This may indicate that soccer players do not periodise their carbohydrate intake during training based on the intensity of the session and may choose to limit carbohydrate intake during training to meet other individual goals. Nevertheless, these findings are limited to only one study. Additional research is required to understand the contemporary carbohydrate intake of soccer players during training in cool environments at a high intensity.

Therefore, the aim of this study was to investigate sweat rates, sweat [Na^+^], fluid and carbohydrate intake in elite soccer players during high intensity training in a cool environment. We hypothesised based on the increased training intensity that the soccer players in the current study would display higher sweat rates and sodium loss compared to previous investigations. We also hypothesised that players would experience various magnitudes of hypohydration, with some exceeding 2% of pre-training body mass. Last, we hypothesised that carbohydrate intake during training would be below 30 g per hour.

## 2. Materials and Methods

### 2.1. Study Participants

Thirty-six elite professional soccer players from Wrexham Association Football Club (A.F.C) (English second division; Championship League) completed the study. Thirty-three of these were outfield players and three were goalkeepers. All players completed a health screening questionnaire and provided written informed consent to participate after the details of the study had been explained. The study was approved by the Research Ethics Committee of Loughborough University, U.K. (LEON19816). All players were professional, accustomed to training and/or match durations between 60 and 120 min 3–6 times per week. The physical characteristics of the players were age = 27.6 ± 4.1 years; body mass = 84.6 ± 7.1 kg; stature = 183.9 ± 6.8 cm.

### 2.2. Experimental Design

The measurements were made on four separate training days during the competitive seasons 2024–2025 and 2025–2026. The four training sessions were similar in terms of total distance covered (6890 m vs. 7700 m vs. 6439 m vs. 7681 m) and intensity (m·min^−1^; 102 m vs. 107 m vs. 97 m vs. 117 m). Furthermore, all sessions followed a similar training pattern which included warm-up, followed by technical and tactical drills, and concluded with matchplay. The study design was observational. The results are descriptive only of those training days on which the tests were completed. Training began at approximately 11.00 a.m. and lasted 70 ± 4 min. Each training session was completed outside on a grass pitch. Environmental temperature and relative humidity were recorded (Kesteral 4500, Nielsen-Kellerman, Boothwyn, PA, USA) at the beginning and at 15 min intervals during training. The training was that which was normally conducted by the players at that time of the season, with no interference from the research team. Furthermore, players were free-living with no research-requested dietary controls.

In total 36 players were assessed across four training sessions, with each player being assessed only once. The number of players assessed varied across the four training sessions: 24 players in training session 1; 6 players in training session 2; 3 players in training session 3; 3 players in training session 4. The training intensity was classified by a global positioning satellite system (STAT Sports Group Ltd, Chicago, Illinois, USA.) in outfield players only. The intensity of training is presented in Table 1.

Data were collected under the following conditions classified by ambient temperature (11.3 ± 2.1 °C), wet bulb globe temperature (9.8 ± 3.0 °C), and relative humidity (76.8 ± 10.2%). Testing in the four training sessions took place in Wrexham, Wales (November-March) during two competitive seasons (2024–2025 and 2025–2026). The testing schedule was dictated by player availability during international breaks throughout the competitive season and during the winter break. Players self-selected the clothing worn on each testing occasion. Outfield players wore a training shirt, shorts, soccer socks, base layers that covered limbs and torso, neck scarves, and soccer boots. During the training sessions, all players had free access to water, a 6% carbohydrate–electrolyte beverage (Gatorade Thirst Quencher, PepsiCo) and an electrolyte beverage (Hydro Electrolyte Tablets, Science in Sport), all in individually labelled drinks bottles. Players were allowed to drink between drills.

### 2.3. Assessment of Fluid Balance

A pre-exercise urine sample was collected from players 30–60 min prior to exercise. These samples were analysed for urine-specific gravity (USG) (Atago 3730 Pen-Pro Dip-Style Digital Refractometer, Washington, DC, USA) to provide an indication of pre-training hydration status. Hydration status was classified as follows: <1.020 euhydrated, 1.021–1.024 minimally hypohydrated, >1.024 hypohydrated [26]. Containers were allocated to each player to collect any urine passed during training. In the hour before training, players were encouraged to void their bladder before body mass (kg) was recorded in minimal clothing. Sweat loss was calculated from the change in body mass collected after exercise, following the correction for fluid intake and any urine/stool loss [27]. Relatively small changes in mass due to substrate oxidation and other sources of water loss were ignored [28].

### 2.4. Assessment of Sweat [Na^+^]

Full details of the sweat collection protocol have been described in detail by Baker and colleagues (2014). In summary, absorbent sterile patches (Tegaderm + Pad, 3M, Loughborough, UK) were applied to specific landmarks on the surface of the thigh and upper back on the right side of the body. The patches were applied prior to the training session and remained in place for the entire duration of the session. These were checked for saturation by the research team during the hydration breaks. No patches were removed before the end of training due to early saturation. In all 36 players across all four training sessions, the upper back patch was analysed with no exceptions. The sweat samples were analysed with a compact wireless analyser that uses ion-selective electrode technology to derive measures of [Na^+^] (Horiba B-722) [29]. This device was previously validated for measuring sweat Na^+^ during exercise [29]. The device was calibrated with two known Na^+^ concentrations (150 & 2000 ppm sodium standards) before utilisation and sample analysis. Regional sweat [Na^+^] was normalised to whole-body concentrations following the prediction equations developed by Baker et al. [29]. Equivalent sodium chloride losses per hour of exercise were calculated from the sweat [Na^+^] and individual sweat rate [27].

### 2.5. Statistical Analysis

Data are reported as mean and standard deviation (mean, s), with the range of data given in parentheses. All data were analysed using SPSS v28 (IBM, Armonk, NY, USA). Data from all sessions were pooled together. Where GPS data were missing, participants were excluded from correlation comparisons. A Shapiro–Wilk test was used to determine normality of distribution with Pearson Correlation Coefficient or Spearman Rank Correlation performed (depending on whether data were normally or not normally distributed) to determine the strength of the relationships between sweat rate and sodium loss, respectively, and metrics of training intensity for outfield players (*n* = 30). Statistical significance was declared when *p* < 0.05. Differences between playing positions were not assessed due to being underpowered. However, data are displayed based on the playing position in the Results section.

## 3. Results

The physical performance of the players during the training sessions was classified as high-intensity exercise by the parameters listed in Table 1, and in line with previous literature [11,13]. Pre-exercise urine was collected in eleven out of the thirty-six players that completed the assessment. The remaining players were not able to provide a pre-exercise urine sample either because they were not able to urinate or due to a lack of time based upon training logistics. Two of the eleven players for which USG was collected were classified as ‘minimally hypohydrated’ (1.022 and 1022, respectively), while the remaining nine players had urine-specific gravity indicative of ‘euhydration’ (<1.020).

**Figure 1:**
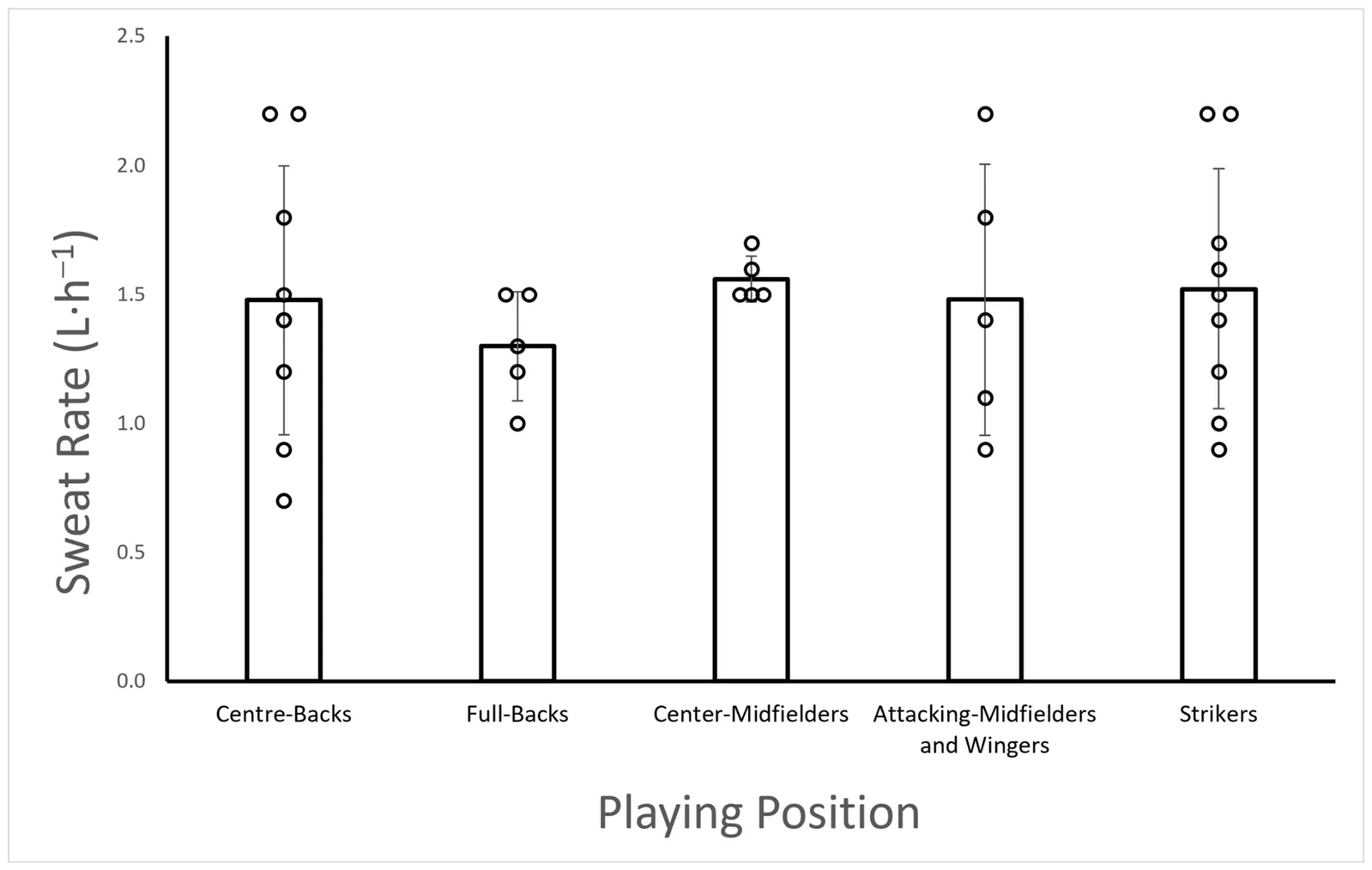
Sweat rate (L·h^−1^) for outfield players based on playing position: Centre-backs (*n* = 9); Full-backs (*n* = 5); Centre-midfielders (*n* = 5); Attacking-midfielders and Wingers (*n* = 5); Strikers (*n* = 9). Values are presented as means ± SD. Points represent individual values for each player.

**Figure 2:**
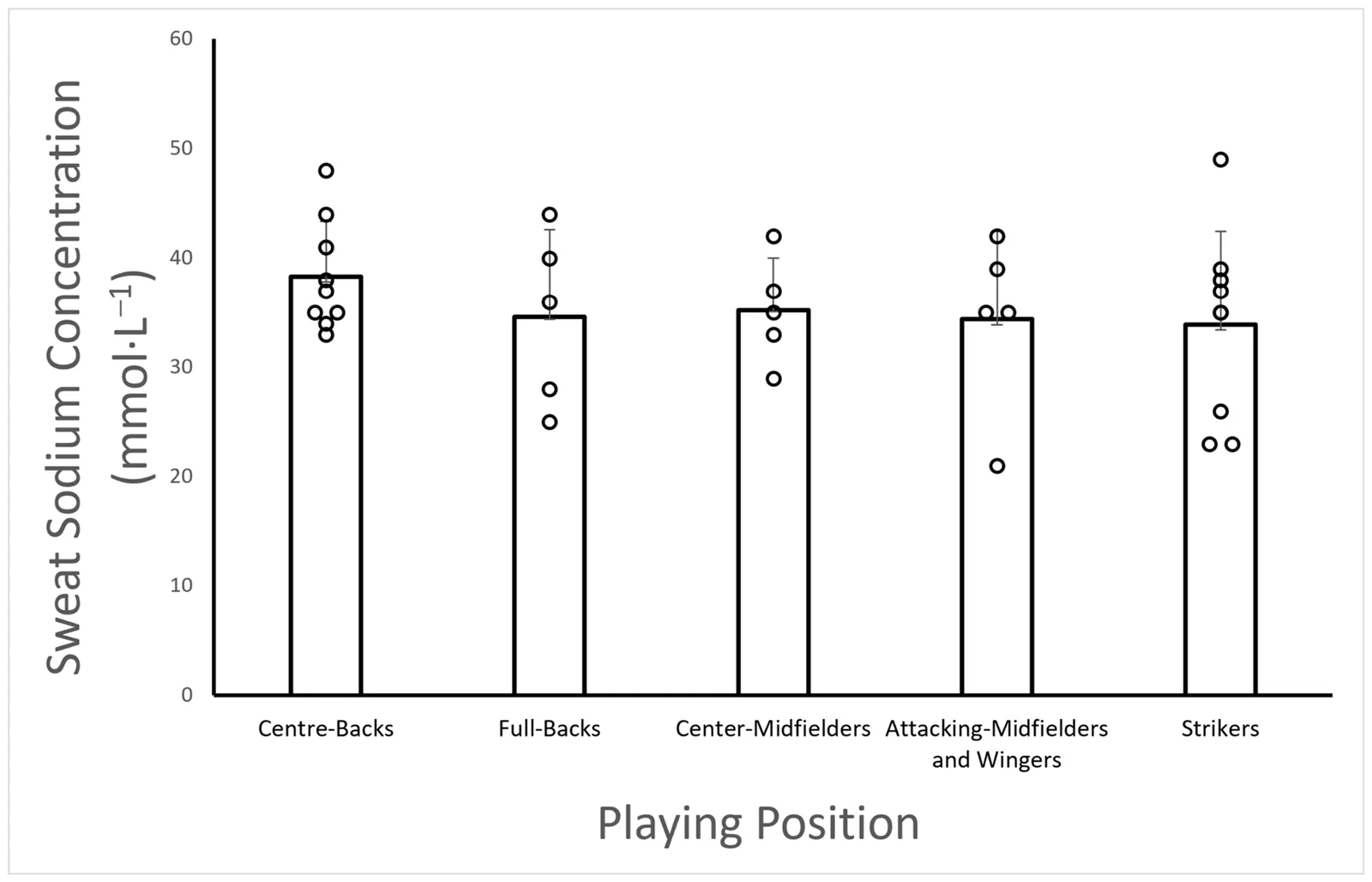
Sweat sodium concentration (mmol·L^−1^) for outfield players based on playing position: Centre-backs (*n* = 9); Full-backs (*n* = 5); Centre-midfielders (*n* = 5); Attacking-midfielders and Wingers (*n* = 5); Strikers (*n* = 9). Values are presented as means ± SD. Points represent individual values for each player.

The fluid balance variables for the outfield players (*n* = 33) in the four training sessions are reported in Table 2. Mean sweat rate for outfield players was 1.5 ± 0.4 (0.4–2.2) (L·h^−1^). Mean and individual sweat rate (L·h^−1^) for outfield players are presented based on playing position in Figure 1. Mean fluid intake in this group of players was 0.66 ± 0.37 (0.07–1.40) (L·h^−1^). Mean change in body mass was −1.1 ± 0.6 (−2.5–0.4) (kg), which was the equivalent of a mean percent body mass change of −1.3 ± 0.7 (−2.7–0.5) (%). Six outfield players lost > 2% of pre-training body mass. Mean sweat [Na^+^] in this group was 35 ± 6.8 (21–49) (mmol·L^−1^). Mean and individual sweat [Na^+^] for outfield players are presented based on playing position in Figure 2. Mean sodium chloride loss was 2.9 ± 1.3 (0.9–6.4) (g·h^−1^). The carbohydrate intake is reported in Table 2. The frequency of players ingesting 0 g of carbohydrate during the four training sessions was *n* = 23 out of *n* = 36. Mean carbohydrate intake across all outfield players was 6 ± 9 (0–25), whilst mean carbohydrate intake only in players that consumed carbohydrate was 15 ± 7 (3–25). Urine was produced by four players across the training sessions, with mean urine output: 0.03 ± 0.09 L.

Mean sweat rate for goalkeepers (*n* = 3) was 0.7 ± 0.2 (0.6–0.9) (L·h^−1^). Mean fluid intake in this group of players was 0.30 ± 0.27 (0.01–0.53) (L·h^−1^). Mean change in body mass was −0.6 ± 0.5 (−0.2–1.1) (kg), which was the equivalent of a mean percent body mass change of −0.6 ± 0.6 (−0.2–1.3) (%). Sweat [Na^+^] was 25 mmol·L^−1^ (*n* = 1), while sodium chloride loss was 0.7 g·h^−1^ (*n* = 1). Mean carbohydrate intake for goalkeepers (*n* = 3) was 1 ± 1.7 g·h^−1^.

There were no significant correlations between sweat rate and total distance covered (r_s_ = 0.097, *p* = 0.615), intensity of play (m·min^−1^) (r_s_ = 0.247, *p* = 0.196), high-speed running (r_s_ = −0.157, *p* = 0.417), and number of sprints (r_s_ = −0.176, *p* = 0.362).

**Figure 3:**
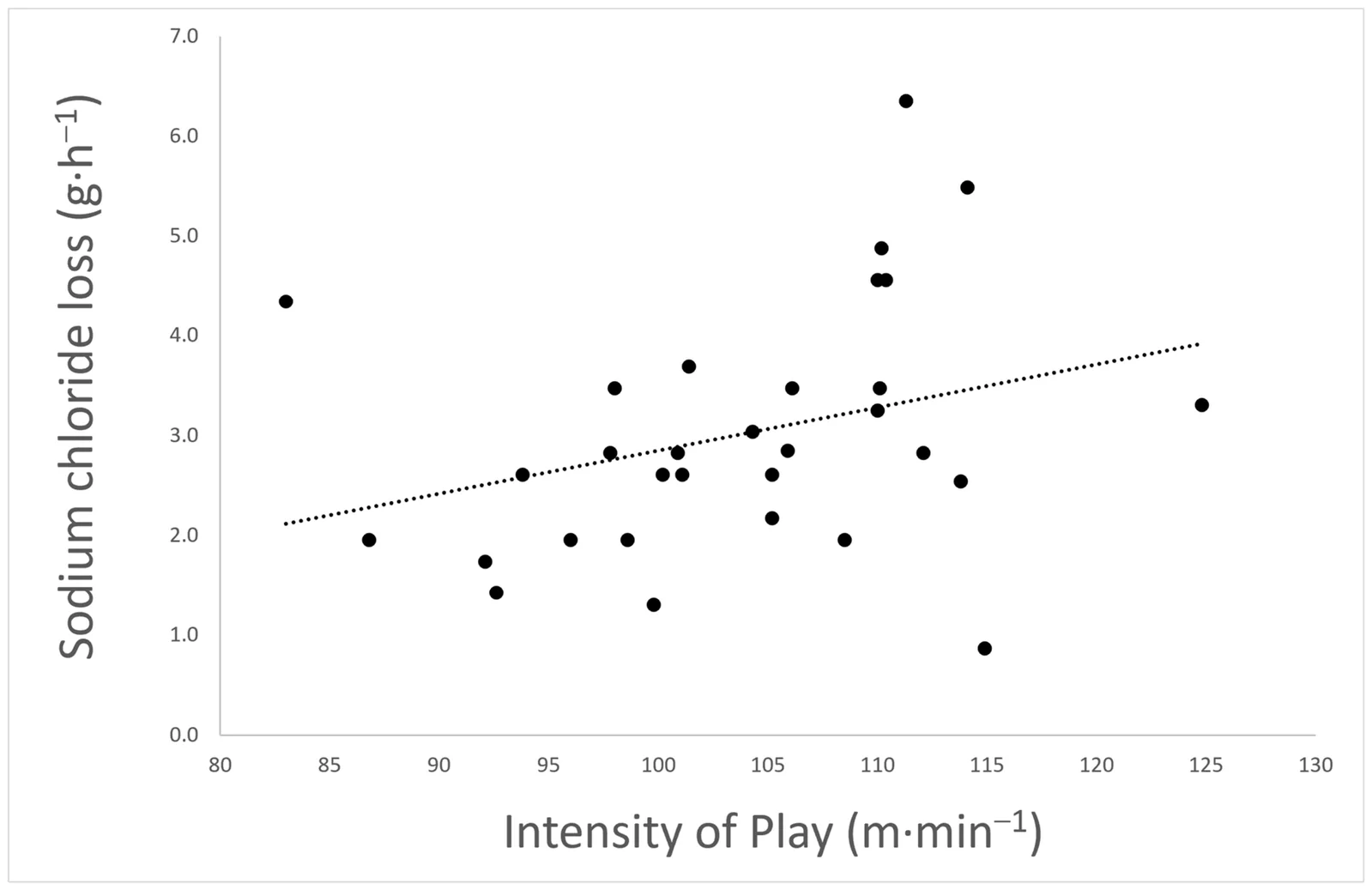
Relationship between sodium chloride loss (g·h^−1^) and intensity of play (m·min^−1^) covered during training (r_s_ = 0.368, *p* = 0.049). Points represent individual values for each player.

There was a significant positive correlation between sodium chloride loss per hour and intensity of play (m·min^−1^) covered during training (Figure 3, r_s_ = 0.368, *p* = 0.049). However, there were no significant correlations between sodium chloride loss per hour and total distance (r_s_ = 0.259, *p* = 0.175), high-speed running (r_s_ = −0.307, *p* = 0.106), and sprints (r_s_ = −0.239, *p* = 0.211).

## 4. Discussion

The key findings of the present study were as follows: (1) six players experienced hypohydration > 2% of pre-exercise body mass; (2) sweat rate and sweat [Na^+^] varied largely between individuals; (3) there was a significant positive correlation between sodium chloride loss per hour and metres covered per minute; (4) carbohydrate ingestion during training varied between individuals and was below recommended intake for a soccer training session of this intensity and duration.

The mean sweat rate for outfield soccer players was higher (1.5 L·h^−1^) compared to previous findings (1.0–1.1 L·h^−1^) when training in cool environments [10,11,13]. The environmental conditions were similar (11 °C & 77% RH vs. 5–15 °C & 66–81% RH) between the present and previous studies. Furthermore, the duration of the training sessions in the present study (70 ± 4 min) were similar to previous studies (65–90 min) completed in cool environments [10,11,13]. The soccer players in the present study competed in the same league (English Football League Championship) as the players in the Sabou et al. (2020) [13], whilst the other two studies completed in cool environments [10,11] included soccer players competing in the Dutch and Spanish First Division, respectively. Based on the similar environmental conditions, players’ calibre and session duration, the higher sweat rates identified in the current study are likely a consequence of the total distance covered and intensity of training. The mean total distance covered was higher compared to a previous study, in which players completed field-based training in a cool environment (7054 m vs. 4889 m) [11]. Furthermore, a subsequent key difference is that on average players covered 104 m·min^−1^ as opposed to 82 m·min^−1^ [11]. Furthermore, a greater distance was covered at high running speeds in the current study (543 m vs. 232 m vs. 291 m) in comparison to previous investigations in cool environments [11,13].

There were no significant correlations between sweat rate and running parameters, including total distance covered, metres covered per min, high-speed running and number of sprints. A significant correlation between sweat rate and total distance covered during soccer training has been reported previously [13] However, similar to the present study, no correlation was reported between sweat rates and intensity, as indicated by the distance covered at high speed or number of high-intensity efforts [13]. This is surprising given exercise intensity is an established variable contributing to sweat rates during soccer training [11]. An immediate explanation for the lack of correlation is unclear. However, this may be a consequence of the more homogeneous sweat rates between players compared to the wider range observed in their sweat sodium concentration (Table 2).

Goalkeepers’ mean sweat rate was markedly lower compared to the sweat rate of outfield players (0.7 vs. 1.5 L·h^−1^). However, importantly no data were collected for GPS metrics or heart rate as part of the sessions, which prevented an accurate estimation of the goalkeeper training intensity. More research is required on fluid balance in goalkeepers, who are an under-reported cohort in elite male soccer [31]. Nevertheless, it appears that based on the low sweat rates identified and their on-going access to fluids during soccer matches, goalkeepers are less likely to be at risk of achieving dehydration levels that may impair their performance.

Fluid loss is of interest in professional soccer, due to the reduction in both physical and technical performance when hypohydrated, particularly at levels above 2% of pre-exercise body mass [15,16]. Several players (*n* = 6) experienced hypohydration > 2% of pre-exercise body mass. The greatest body mass loss (2.7%) in the current study, closely matches the greatest value reported previously (2.5%) in soccer players training in cool conditions [10]. However, our findings contrast with studies which report that hypohydration rarely exceeds 2% of pre-exercise body mass in soccer players training in cool environments [11,13]. The discrepancy between the current and previous studies is likely driven by the aforementioned differences in training intensity, but also the different hydration and fuelling strategies employed. This is important particularly in the context of soccer matches, where total distance covered (10–13 km vs. 7 km), high-speed running distance (>900 m vs. 500 m) and sprint distance (>200 m vs. 100 m) [23] are higher compared to the present study, whilst access to fluids may be reduced. Combined, these factors can further increase dehydration to levels where physical and technical soccer performance may be negatively impacted [15,16].

The carbohydrate intake of elite soccer players during training was only previously investigated in a limited number of studies [11,32]. We identified a mean carbohydrate intake of 6 g·h^−1^. This finding is similar to the value reported by Anderson et al. (3 g·h^−1^) for soccer players during training [32], but slightly lower than the one reported by Rollo et al. in soccer players training at high-intensity in cool environments (11 g·h^−1^) [11]. However, when looking only at the players that consumed carbohydrates during training, the mean intake was 15 g·h^−1^, which is similar to the intake identified by Rollo et al. [11]. A common finding is that carbohydrate intake varies between players completing the same training session. Nevertheless, carbohydrate intake during training is consistently below the intake reported during match-play in elite soccer players (32 g·h^−1^) [32]. Interestingly, despite availability, 23 out of the 36 players chose not to consume any carbohydrates during training. For 60–70 min of intense training, ingesting 30–60 g of carbohydrates per hour may benefit physical and technical components of soccer performance [33,34], although this may be influenced by their pre-exercise dietary intake [35]. Furthermore, consuming carbohydrate during training sessions which are close to match-play intensities may also ‘train’ the gut [22]. Nonetheless, all players completed the training sessions without volitional fatigue or noticeable declines in skill execution. Thus, the implications of not meeting carbohydrate intake recommendations during training sessions per se remain to be determined. Furthermore, soccer players may deliberately choose not to consume carbohydrate during training to help meet individual body composition goals and/or daily carbohydrate targets [23]. Alternative reasons for which soccer players may choose not to consume CHO during training include adopting a ‘train low’ approach for promoting fat oxidation adaptation [24], as well as chronically adhering to a ketogenic diet [25]. Future studies should investigate the key influencing factors that drive carbohydrate consumption (or lack of) during training in elite soccer players.

The mean and range of sweat [Na^+^] values (35 mmol L^−1^; 21–49 mmol·L^−1^) are consistent with several previous studies observing soccer players training in cool environments [10,11]. Furthermore, 16 out of the 36 players observed in this study displayed sweat [Na^+^] values that are classified as ‘moderate’ (36–58 mmol·L^−1^), with all remaining players’ values classified as ‘low’ (<36 mmol·L^−1^) [36]. The equivalent sodium chloride losses (2.9 g·h^−1^) were slightly higher compared to previous investigations (2.2–2.6 g·h^−1^) conducted in elite soccer players training in a cool environment [10,11,13]. This was predominantly driven by the higher sweat rates observed in the present study, rather than the sweat [Na^+^] values, which were consistent with the previous literature [10,11].

### 4.1. Study Limitations and Future Research

The present study was descriptive and therefore several limitations should be acknowledged. Firstly, the data was collected across four different training sessions. Nonetheless, the GPS data collected shows that all four training sessions were of similar intensity based on classification thresholds. Second, the sweat analysis completed did not include potassium concentration, which is useful for quality control for sweat samples [37]. Despite this, best practices of sweat collection (clean skin, avoiding patch saturation, gloves, etc.) described by Baker [37] were followed. Therefore, we have confidence in our analysis. Due to the descriptive design of the study, factors such as hormones [38] and diet [39] that have previously been reported to influence sweat [Na^+^] were not controlled for. The limitations of USG for hydration assessment have been described previously [40]. However, more invasive measures, such as measuring plasma osmolality, were not possible in the present study. Future studies should include additional measures such as first-morning USG, 24 h urine volumes, and/or 24 h fluid intake prior to training to provide further insights into the influence of baseline hydration on sweat rates and sweat [Na^+^].

### 4.2. Practical Applications

The findings of this study indicate that the *ad libitum* hydration strategies employed by elite male soccer players training at high intensity in a cool environment may not be sufficient to prevent hypohydration of <2% pre-exercise body mass in all players. Furthermore, our data re-confirms that sweat rates and sweat [Na^+^] are variable in elite soccer players. Importantly, sweat testing data can be collected in this population without disruption to normal training or competitive schedules. This study also identified a significant, albeit modest correlation between the metres per minute covered by soccer players and their sodium chloride loss. Practitioners can utilise the present data to inform the hydration recommendations for elite soccer players when training at high intensity in cool environments. This is important, as hydration may be perceived as less important in cool environments than during training in the heat.

## 5. Conclusions

In conclusion, sweat rate and sweat sodium loss varies in elite soccer players during high-intensity training in cool environments. Hypohydration exceeded 2% pre-exercise body mass in 17% of players. Despite the duration and high-intensity nature of the training sessions, the majority of soccer players (23/36) in the present study chose not to consume any carbohydrates.

## Figures and Tables

**Table 1:** Global positioning satellite parameters for training intensity classification for pooled training sessions. High-speed running defined as <5.5 m·s^−1^ and <7 m·s^−1^. Sprint defined as >7 m·s^−1^.

Running Parameter	
Total distance (m)	7054 ± 654
Intensity of play (m·min^−1^)	104 ± 9
High speed running (m)	543 ± 141
Number of sprints	6 ± 3

**Table 2:** The mean ± s (range) of variables recorded before and during each training session in response to exercise at high intensity in a cool environment. Whole-body sweat [Na^+^], and equivalent whole-body sweat sodium chloride losses during exercise. The loss of sodium chloride is calculated on the assumption that all Na^+^ loss is sodium chloride [30].

	Outfield Players (*n* = 33)
Urine-specific gravity (USG)	1.010 ± 0.007 (1.003–1.022)
Sweat rate (L·h^−1^)	1.5 ± 0.4 (0.4–2.2)
Fluid intake (L·h^−1^)	0.66 ± 0.37 (0.07–1.40)
Change in body mass (kg)	−1.1 ± 0.6 (−2.5–0.4)
Percent body mass change (%)	−1.3 ± 0.7 (−2.7–0.5)
Number of players that lost > 2% of pre-training body mass	6
Sweat [Na^+^] (mmol·L^−1^)	35 ± 6.8 (21–49)
NaCl loss (g·h^−1^)	2.9 ± 1.3 (0.9–6.4)
Carbohydrate intake (g·h^−1^) (a) All players (*n* = 36) (b) Players that consumed carbohydrates (*n* = 13)	(a) 6 ± 9 (0–25) (b) 15 ± 7 (3–25)

## Data Availability

The data presented in this study are available on request from the corresponding author and the permission of all parties involved in the study. The data are not publicly available due to privacy.
